# Whole-Genome DNA Methylation Analysis of Peripheral Blood Mononuclear Cells in Multiple Sclerosis Patients with Different Disease Courses

**Published:** 2016

**Authors:** O.G. Kulakova, M.R. Kabilov, L.V. Danilova, E.V. Popova, O.A. Baturina, E.Y. Tsareva, N.M. Baulina, I.S. Kiselev, A.N. Boyko, A.V. Favorov, O.O. Favorova, V.V. Vlassov

**Affiliations:** Pirogov Russian National Research Medical University, Ostrovityanova str. 1, Moscow, 117997, Russia; Institute of Experimental Cardiology, Russian Cardiology Scientific and Production Center, 3th Cherepkovskaya str. 15A, Moscow, 121552, Russia; Institute of Chemical Biology and Fundamental Medicine, Siberian Branch of the Russian Academy of Sciences, Lavrentiev Avenue 8, Novosibirsk, 630090, Russia; Vavilov Institute of General Genetics, Russian Academy of Sciences, Gubkina str. 1, Moscow, 119991 , Russia; Johns Hopkins School of Medicine, Baltimore, MD 21205, USA

**Keywords:** epigenetics, DNA methylation, multiple sclerosis, peripheral blood mononuclear cells

## Abstract

Multiple sclerosis (MS) is a severe neurodegenerative disease of polygenic etiology affecting the central nervous system. In addition to genetic factors, epigenetic mechanisms, primarily DNA methylation, which regulate gene expression, play an important role in MS development and progression. In this study, we have performed the first whole-genome DNA methylation profiling of peripheral blood mononuclear cells in relapsing-remitting MS (RRMS) and primary-progressive MS (PPMS) patients and compared them to those of healthy individuals in order to identify the differentially methylated CpG-sites (DMSs) associated with these common clinical disease courses. In addition, we have performed a pairwise comparison of DNA methylation profiles in RRMS and PPMS patients. All three pairwise comparisons showed significant differences in methylation profiles. Hierarchical clustering of the identified DMS methylation levels and principal component analysis for data visualization demonstrated a clearly defined aggregation of DNA samples of the compared groups into separate clusters. Compared with the control, more DMSs were identified in PPMS patients than in RRMS patients (67 and 30, respectively). More than half of DMSs are located in genes, exceeding the expected number for random distribution of DMSs between probes. RRMS patients mostly have hypomethylated DMSs, while in PPMS patients DMSs are mostly hypermethylated. CpG-islands and CpG-shores contain 60% of DMSs, identified by pairwise comparison of RRMS and control groups, and 79% of those identified by pairwise comparison of PPMS and control groups. Pairwise comparison of patients with two clinical MS courses revealed 51 DMSs, 82% of which are hypermethylated in PPMS. Overall, it was demonstrated that there are more changes in the DNA methylation profiles in PPMS than in RRMS. The data confirm the role of DNA methylation in MS development. We have shown, for the first time, that DNA methylation as an epigenetic mechanism is involved in the formation of two distinct clinical courses of MS: namely, RRMS and PPMS.

## INTRODUCTION

Multiple sclerosis (MS) is a severe neurodegenerative disease of the central nervous system (CNS) characterized by a complex combination of pathogenetic processes in which the most important role belongs to a chronic autoimmune inflammation directed against the components of the myelin sheath of neurons and resulting in demyelination, loss of oligodendrocytes, destruction of axons, gliosis, and neurodegeneration. The etiology of MS remains unclear. Recent whole-genome studies have clearly demonstrated that the observed mode of MS inheritance, typical for polygenic diseases, is indeed defined by the joint contribution of many independently acting or interacting polymorphic genes [1-3]. However, if one excludes the genes of the major histocompatibility complex (HLA) from consideration, each of the remaining MS risk alleles, taken separately, is associated with a relatively small effect: odds ratios for individual single nucleotide polymorphisms (SNPs) are, with few exceptions, in the range of 1.1–1.3 [4]. The joint contribution of all the genetic variants identified in whole-genome studies explains less than 27% of heritability [5]; a problem known as “missing heritability.” These observations, as well as the low level of MS concordance in monozygotic twins [6], the effect of some environmental factors [7] and a higher prevalence of MS among women [8], led to an assumption that, in addition to genetic factors, epigenetic mechanisms may play an important role in MS development and progression.

Epigenetic modifications are various functional changes in the genome that affect the expression of genes in different cells or tissues, but are not associated with changes in the DNA sequence. DNA methylation is one of the best studied epigenetic mechanisms, and its most common form involves the addition of a methyl group to the C5 position of a cytosine ring in CpGdinucleotides. This process modulates the expression of nearby genes. Although global DNA methylation is a relatively stable epigenetic modification, which is passed onto daughter cells during the mitosis, various environmental factors can cause dynamic changes in the epigenome during a lifetime. Recent results indicate that epigenetic modifications may play an important role in shaping the risk of autoimmune and neurodegenerative diseases, particularly MS [9, 10].

A comparative analysis of gene-specific methylation in MS patients and healthy donors revealed hypomethylation of the promoter region of the *PAD2 *gene encoding type II peptidyl arginine deiminase in the white matter of the brain [11] and in peripheral blood mononuclear cells (PBMCs) [12]. Also, the *SHP-1 *gene encoding protein tyrosine phosphatase was identified as hypermethylated in PBMCs of MS patients [13]. Several comparative whole-genome studies of DNA methylation profiles in MS patients and healthy individuals have been conducted in the past five years. The methylome was analyzed in both the blood cells (PBMC, CD4+, CD8+ T-lymphocytes) [6, 14-16] and the white matter of the brain [17]. Even though all studies were conducted on small sets of samples, it was found that HLA class II genes and some other immune system genes whose association with the disease had been demonstrated previously were differentially methylated in CD4+ T-lymphocytes [15] and differentially methylated genes associated with survival of oligodendrocytes were identified in the white matter of the brain [16].

MS is characterized by pronounced clinical heterogeneity [8]. The cited papers focused on patients with the most common form of MS, relapsing-remitting MS (RRMS), which is characterized by alternating periods of exacerbation and remission. Approximately 10–15% of patients are suffering from primary progressive MS (PPMS) that manifests as a continuous increase in the neurological deficit from the onset of the disease. The course of PPMS is much more severe than that of RRMS; signs of brain atrophy can be clearly defined already in the early stages of PPMS. To date, there is no specific treatment for PPMS patients and all currently known immunomodulatory drugs and corticosteroids that are used to treat RRMS are ineffective in this case.

In this paper we have conducted the first genomewide analysis of the DNA methylation profile in PBMCs of patients with RRMS and PPRS in comparison with a control group of healthy individuals in order to identify differentially methylated CpG-sites (DMSs) associated with the development of the two major clinical disease courses, and compared the profiles of DNA methylation in patients with RRMS and PPMS.

## MATERIALS AND METHODS

**Characteristics of MS patients and controls**

The study included 14 patients with RRMS (9 women and 5 men) and 8 patients with PPRS (6 women and 2 men), aged 29 to 58 years. The diagnosis was established according to the latest version of the McDonald criteria from 2010 [18]. The average score on the EDSS disability scale was 2.32 ± 0.823 for RRMS patients and 4.29 ± 0.39 for PPMS patients. The patients included in the study had never received any immunomodulatory drugs. The control group included 6 women and 2 men, aged 28 to 50 years, without acute or chronic neurological diseases. They all lived in the Moscow region; both of their parents were ethnic Russians (according to the survey). All participants provided written informed consent for genetic research.

**DNA isolation and whole-genome analysis of methylation**

Samples of the peripheral blood from MS patients (8 ml) were collected in tubes containing EDTA (Vacuette ® EDTA Tubes, Greiner Bio-One). The peripheral blood mononuclear cells (PBMC) were isolated by centrifugation on a Ficoll-Hypaque gradient. The genomic DNA was isolated using the DNA Midi Kit (Qiagen, Santa Clara, CA, USA) according to the manufacturer’s procedure. Bisulfite conversion of the genomic DNA was performed using the EZ DNA Methylation-Gold Kit (Zymo Research). The level of DNA methylation was analyzed using a iScan scanner (Illumina) and Infinium HumanMethylation450 BeadChip [19] at the SB RAS Genomics Core Facility (ICBFM SB RAS).

**Bioinformatic analysis**

Primary data processing and normalization were performed using specially developed scripts written in R programming language [20].

The assessment of the methylation level for each CpG-site in the sample was performed by calculating a beta-value that is a ratio of the intensity of the methylated signal to the total intensity of the probe (sum of intensities of methylated and unmethylated signals). Beta-values ranged from 0 (unmethylated probe) to 1 (fully methylated probe). The *methylumi *package was used to calculate beta-values for each probe in each sample [21].

The probes containing a single nucleotide polymorphism within 10 bp of the interrogated CpG-site and probes, which overlapped with repeat DNA elements within 15 bp of the interrogated CpG-site, were excluded from the subsequent analysis. The probes with a detection *p*-value greater than 0.05 in more than 5% of the samples and probes located on the X and Y chromosomes were also excluded. As a result, a total of 384,138 CpG-sites out of the initial 485,000 were analyzed.

A CpG-site is considered to be differentially methylated if the difference between the methylation levels in two groups fulfills two conditions: the absolute mean difference of beta-values between groups >0.1, and the corresponding *p- *value < 0.01. The localization of an individual CpG-site in a CpG-island was determined using UCSC annotation, version hg19; the CpGshore was located 2 kbp distant from CpG-islands; and the CpG-shelf, – 2 kbp away from the CpG-shore [19]. The *P*-value was estimated using the empirical Bayes modified *t*-test, as implemented in the *limma *package in R [22]. The small sample size precluded performance of adjustment for the number of hypotheses (probes).

Visualization of DMS signals using the principal component analysis (PCA) and heatmap analysis were performed using R standard methods and customized routines [22].

## RESULTS

In order to examine the potential involvement of the epigenetic DNA methylation mechanism in the development of different MS clinical courses, we had carried out a whole-genome profiling of the DNA methylation sites in PBMC from representative groups of patients with RRMS and PPMS and healthy donors (controls) and performed the following pairwise comparisons: RRMS patients vs. the controls, PPMS patients vs. the controls, and RRMS patients vs. PPMS patients.

**Fig. 1:**
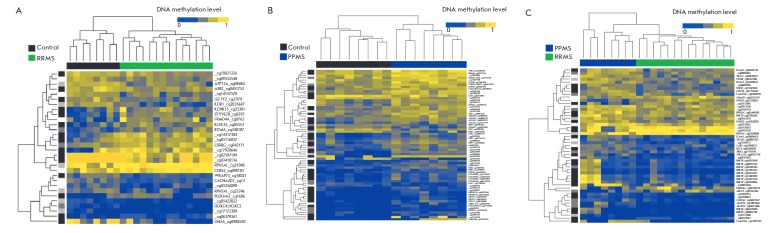
Heatmaps of differentially methylated sites of peripheral blood mononuclear cells in RRMS patients vs. the healthy group (A), PPMS patients vs. healthy group (B), and PPMS patients vs. RRMS patients (C).** Top panel**. Dendrogram showing the results of hierarchical sample clustering. Green color indicates DNA samples from RRMS patients; blue color, from PPMS patients; black color, from healthy individuals.** Left panel**. Dendrogram showing the results of hierarchical DMS clustering. The intensity of grey indicates DMS localization in the genome (black color, CpG-islands, dark grey, CpG-shores, light gray, CpG-shelves).** Right panel. **Samples labeling based on Human Methylation450BeadChip standard annotation. RRMS, relapsing-remitting multiple sclerosis; PPMS, primary progressive multiple sclerosis.

Heatmaps of the DMSs identified in these three types of pairwise comparisons are shown in *Fig. 1A,B*. A total of 136 DMSs were identified for one or more pairwise comparison. Comparative analysis of DNA methylation for all three pairwise comparisons revealed significant differences in the methylation profiles. Hierarchical clustering by the methylation level of 136 identified DMSs revealed a clearly defined aggregation of DNA samples from patients of each of the groups under study into separate clusters. A visual comparison of the intensity of DMSs signals (ratio of blue and yellow) indicates a higher level of DNA methylation in PPMS patients compared to the controls and RRMS patients.

**Fig. 2:**
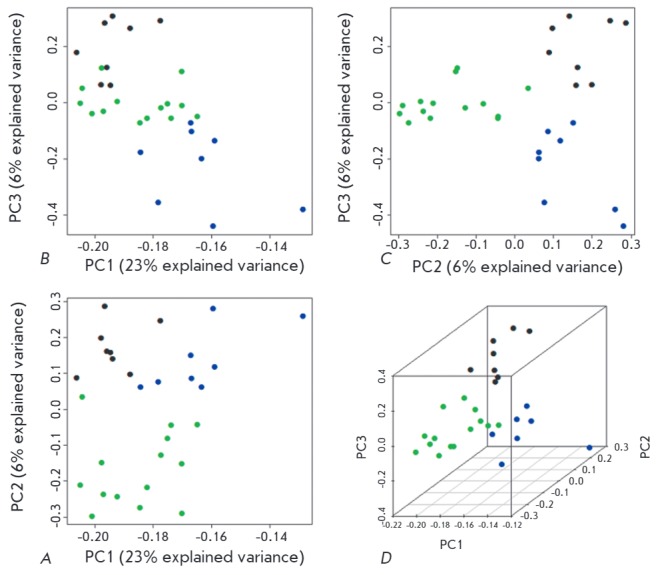
2D (*A–C*) and 3D (*D*) samples clustering based on a principal component analysis (PCA) of differentially methylated sites (DMSs). The green dots indicate RRMS samples; blue dots PPMS samples; and black dots, healthy controls. The axes: the principal components PC1, PC2, and PC3; the proportion of explained variance of the data is indicated in brackets for each principal component.

The data for these DMSs were visualized using the principal component analysis (*Fig. 2*). As follows from the figure, the samples included in the study are well discriminated in a three-dimensional space of the first three principal components into three groups and these groups correspond to three phenotypes: RRMS, PPMS, and absence of these diseases.

**Table 1:** Characteristics of the differentially methylated sites (DMSs) identified in a comparative analysis of DNA methylation of PBMCs from patients with different clinical courses of MS and healthy individuals (controls)

Compared groups	RRMS vs control	PPMS vs control	PPMS vs RRMS
Number of DMSs	30	67	51
Of them, DMSs with a higher methylation level in the first of the two compared groups	13 (43%)	58 (86%)	42 (82%)
Number of DMSs located in the genes	18	38	35
Number of genes containing DMSs	17	25	22
Of them, genes containing DMSs with higher methylation levels in the first of the two compared groups	9 (53%)	19 (76%)	19 (86%)
Protein encoding genes containing DMSs (number of DMSs in the gene)^#^	ASB2 ATP11A CACNA2D3 CERS5 ESRRG FRMD4A GNAS HOXC4-HOXC6 IFITM5 ILDR1 KCNK15 KLHL35 LEFTY2 PLEKHA2 RPH3AL (2)* WRAP73 ZFYVE28	ATG16L2 (3) CES1 CSGALNACT2 (2) CYB5D1;LSMD1 FAM110A GDF7 (4) HKR1 HLA-F HOXB13 IGSF9B (2) ILDR1 LDB2 MTPN;LUZP6 NTN1 OPCML OR2L13 (3) RBM46 TBX1 TCP10L TMEM44 VIPR2 WRAP73	ABCC5 AKAP12 (2) CARS CBFA2T3 CCDC67 FAM110A FRMD4A GIMAP5 HIVEP3 ICAM5 (2) KCNQ1 KLF4 LEFTY2 OLFM3 PTH1R RASA3 RNF39 (11) RPH3AL(2)* TRAF3 USP35 XKR5
Non-protein encoding genes containing DMSs (number of DMSs in the gene)^#^	-	LINC00116 (2) ZNRD1-AS1 LOC441666 (4)	FAM153C

Note. Genes with DMSs with higher methylation levels in the first of the two compared groups are shown in bold.

^*^Two DMSs were identified in the RPH3AL gene: one with a higher methylation level, the other, with a lower one.

^#^Indicates the number of DMSs located in each gene, if more than one.

The characteristics of the identified DMSs are presented in *Table 1*. A comparison of RRMS patients with healthy donors (controls) revealed differences in the methylation levels of 30 DMSs; for PPMS patients vs. the controls there were 67 DMSs, and the comparison of the two clinical courses of MS revealed 51 DMSs. In the case of RRMS, most of the probes were hypomethylated compared with the controls (only 43% of 30 DMS were hypermethylated). In contrast, PPMS patients had a higher number of hypermethylated probes compared with both the controls (86% of the probes were hypermethylated) and RRMS patients (82% of the probes were hypermethylated).

More than half of the DMSs are located in the genes: 18 out of 30 for RRMS vs controls, 38 out of 67 for PPMS vs controls, and 35 out of 51 for PPMS vs RRMS (*Table 1*). Since in the HumanMethylation450 platform the probes that are located in genes account for approximately a third of all probes [19], there is a clear excess of the number of expected intragenic DMSs for a random distribution of DMSs across the probes. Some genes contain several DMSs; therefore, the number of genes with DMSs is lower: 17, 25, and 22, respectively. The lists of genes containing DMSs (protein encoding, which are in the majority, and non-protein encoding genes) are also presented in *Table 1*. When RRMS is compared with the control, 53% of the genes in the first group contain DMSs with a higher methylation level; these values for the PPMS vs the control and PPMS vs RRMS are 76% and 86%, respectively. The *RPH3AL* gene has two DMSs, one of which is characterized by a higher and the other by a lower methylation level for RRMS vs. control and PPMS vs. RRMS comparisons.

According to the criteria adopted in the study, a CpG-site is considered to be a DMS if there is a 10% difference in the average methylation levels between two groups (i.e. absolute mean difference of beta-values between the groups must be > 0.1). Some DMSs are characterized by a significantly higher difference in the methylation level. The absolute mean difference of beta-values for RRMS patients vs. healthy individuals exceeded 20% for five DMSs. Three of these DMSs are located in the genes: the average methylation level of CpG loci cg07629776 (*FRMD4A*) and cg16866567 (*PLEKHA2*) in RRMS patients was 21.5 and 25.1% higher; and of cg09885502 (*GNAS*), 43.5% lower, respectively, compared with the controls. The comparison of PPMS patients with healthy individuals revealed that 6 out of 67 DMSs have an absolute mean difference of beta-values of more than 20%. The highest difference in the methylation level, namely, an increase by 41% was observed for cg11979743 (*FAM110A*), and it was the only one of the six DMSs that was located in a gene. A direct comparison of PPMS and RRMS identified 7 DMSs whose methylation level differed by more than 20% (20.1 to 31.9%). Of these, 5 DMSs were located in three genes: the average methylation level of cg11979743 (*FAM110A*) was 31.9% higher; and of cg01324343 (*ABCC5*), 21.8% lower in the group of PPMS patients compared with RRMS patients. The third gene, *RNF39*, contained 11 DMSs, and the average methylation level of three of them (cg13401893, cg12633154, cg10568066) was 20.1–21.0% higher in PPMS patients.

Analysis of DMS localization showed that more than half of the DMSs are located in CpG-islands and CpG-shores (*Table 2*; see also *Fig. 1*). This distribution roughly corresponds to the proportion of such probes among all probes of the platform [19]. Comparison of RRMS patients with the controls indicates that 50% of DMSs are located in CpG-islands and 10% in the nearby regions, whereas for PPMS patients the proportion increases to 63 and 16%, respectively, and for PPMS vs. RRMS patients, to 53 and 18% (*Table 2*). Therefore, DNA methylation in PPMS affects more functionally important regions of the genome.

**Table 2:** Localization of differentially methylated sites (DMSs) identified in a comparative analysis of DNA methylation of PBMCs from patients with different clinical courses of MS and healthy individuals (controls), regarding the CpG-islands of the human genome

DMS localization	Number of DMS for different comparisons between the groups		
	RRMS vs control	PPMS vs control	PPMS vs RRMS
Any (total DMSs)	30 (100%)	67 (100%)	51 (100%)
In CpG-island	15 (50%)	42 (63%)	27 (53%)
In CpG-shore	3 (10%)	11 (16%)	9 (18%)
In CpG-shelf	6 (20%)	2 (3%)	5 (10%)
In open sea	6 (20%)	12 (18%)	10 (19%)

Note. Localization of individual CpG-sites in the CpG-island was determined using the UCSC-annotation, version hg19; CpG-shore is located up to 2 kbp from the CpG-island; CpG-shelf – up to 2 kbp from the CpG-shore; areas not related to the above three categories are designated as “Open sea.”

The data taken together form a coherent picture: PPMS differs from RRMS in a higher number of changes in DNA methylation patterns. Indeed, PPMS is characterized by a higher number of DMSs in the genome and its (known) coding part and significantly more than half of the DMSs in both the genome and the genes are hypermethylated. In addition, a higher number of DMSs is localized in CpG-islands and CpGshores in PPMS compared with the controls (79%), whereas in RRMS their share is only 60%.

## DISCUSSION

In order to examine when epigenetic mechanisms are involved in the development of clinically different courses of MS, we performed the first whole-genome analysis of DNA methylation in PBMC of patients with two clinical courses of MS, RRMS, and PPMS, and compared their methylation profiles with healthy donors and with each other. Significant differences in DNA methylation profiles were identified: a pairwise comparison of these three groups (14 RRMS patients, 8 PPMS patients, and 8 individuals in the control group) revealed 136 DMSs with a mean difference in beta-values > 0.1 and *p*-values of < 0.01 according to the Student* t*-test. Three-dimensional visualization of these DMSs using the principal component analysis showed that the DNA samples of the patients from each of the groups under study aggregate into a single cluster, indicating a steady involvement of a differential spectrum of DNA methylation into the development of different clinical courses of MS.

The analysis of the DMS spectrum shows that patients with PPMS, which is a more aggressive clinical course of MS than RRMS, differ from RRMS patients in a higher number of changes in the DNA methylation spectrum in comparison with healthy individuals. Moreover, the number of DMSs with a higher level of DNA methylation is higher in PPMS patients than in the control group and in RRMS patients. At the same time, comparison of RRMS patients with the control group reveals hypomethylation of more than half of the DMSs. The only comparison of our results with published data we can perform is for RRMS patients. A significant DNA hypermethylation in CD8+ T-lymphocytes has been previously identified in patients with RRMS [14], while no changes in the overall level of DNA methylation [14] or a slight decrease were observed [15] in CD4+ T lymphocytes and whole blood.

The analysis of the localization of differentially methylated sites in MS showed that more than half of them are located in either CpG-islands or in the flanking regions (up to 2 kbp distant from a CpG-island, the so-called CpG-shore) (see *Table 2*), which indicates a high probability of the functional importance of the observed DNA methylation, which is known to inhibit the expression of some genes.

We used the GeneCards database of human genes [23], US National Center for Biotechnology Information (NCBI) Gene project [24], and a number of other sources to identify the functions of these genes. Comparison of RRMS patients and healthy individuals reveals differences in the methylation of genes that encode the proteins involved in the development of the immune response (*ASB2*, *LEFTY2*, *PLEKHA2*), the metabolism of lipids (*ILDR1*, *CERS5*), vesicular transport (*RPH3AL*, *ZFYVE28*) and ion channels functioning (*ATP11A*, *CACNA2D3*, *KCNK15*), as well as in the regulation of the expression of many genes (*ESRRG*,* HOXC4-HOXC6*). Among them the *ESRRG *gene encoding estrogen-like receptor gamma deserves special attention. This orphan receptor belongs to the family of nuclear receptors, and through direct binding to the promoter acts as an activator of the transcription for several genes, including the gene encoding the main DNA methyltransferase of mammalian somatic cells, DNA methyltransferase 1 (*DNMT1*) [25], and, thereby, controls the level of DNA methylation in the cell.

Our data on the genes that are differentially methylated in PBMC of RRMS patients compared to healthy individuals do not agree with other studies [6, 14-17]. This is not surprising, since to date a comparative analysis of DNA methylation in RRMS patients and healthy individuals has been performed only in cells of the whole blood, CD4+, CD8+ T lymphocytes [6, 14-16], and in the tissues of the white matter of the brain [17]. There was no agreement between the results obtained for different cell and tissue types, either. The discrepancy between the results may be due not only to the use of different populations of cells, but also to different criteria adopted for the definitions of DMS.

The analysis of the functions of the genes that are differentially methylated in PPMS patients compared with healthy individuals showed that among them there is a group of genes whose products are to some extent involved in the development and differentiation of the nervous system (*GDF7*, *MTPN*, *VIPR2*, *NTN1*,* TBX1*), the functioning of opioid receptors (*OPCML*), and metabolism of various xenobiotics, including cocaine and heroin (*CES1*). Methylation of genes with similar functions was not identified in a pairwise comparison of RRMS patients and healthy individuals. In PPMS, differential methylation also affected the genes involved in the development of the immune response (*HLA-F*, *MTPN*, *VIPR2*), regulating the expression of many genes (*HKR1*, *HOXB13*, *LDB2*, *TCP10L*, *TBX1*), as well as processes of autophagy (*ATG16L2*), hemostasis (*FAM110A*), and the functioning of the extracellular matrix (*CSGALNACT2*).

Among the differentially methylated genes in RRMS and PPMS patients in comparison with controls only two matching genes, *ILDR1 *and *WRAP73(WDR8)*, were identified; both of these genes were hypomethylated in patients. The exact functional significance of their products has not been fully elucidated yet, but ILDR1 receptor activity is at least partially associated with lipid metabolism [26], while the WRAP73 protein comprising conservative WD-repeats can form multimeric complexes with other proteins participating in mitosis, signal transduction in the cell [27], and in the formation of cilia [28].

Our work is the first one to identify the genes whose methylation levels are distinguished in patients with the two main clinical courses of MS. The products of these genes are involved in the development and functioning of immune system cells (*HIVEP3*, *GIMAP5*,* TRAF3*), the regulation of the expression of many genes (*AKAP12*, *RASA3*, *CBFA2T3*), degradation processes (*USP35*), and the functioning of the endocrine system (*PTH1R*). Two genes are of particular interest. The first one, *ICAM5*, encodes a dendrite-specific adhesion molecule of the immunoglobulin superfamily, which is involved in the interaction of nerve cells with each other and with the cells of the immune system in CNS [29]. The other one is *RNF39*. Of the 35 DMSs identified by comparing DNA samples from PPMS and RRMS patients and located in the genes, 11 were located in the* RNF39 *gene and had a higher level of DNA methylation in PPMS. For three of these DMSs, the difference in the methylation level exceeded 20%. The *RNF39* gene is located in the HLA class I gene region. *RNF39* function is not yet clear; however, the association of the polymorphisms of this gene with MS [30] and some other autoimmune diseases [31] has been demonstrated previously. Hypermethylation of the gene in CD4+ T lymphocytes was also found in patients with systemic lupus erythematosus [32].

Our results of a whole-genome analysis of the DNA methylation profiles in PBMCs of MS patients indicate that DNA methylation, one of the main mechanisms of transmission of epigenetic information in mammals, plays a role in the development of MS. It has been demonstrated for the first time that epigenetic DNA methylation is involved in the formation of clinically distinct forms of MS, RRMS, and PPMS, and, in the case of PPMS, methylation, apparently, leads to inhibition of the expression of a higher number of genes.

## Abbreviations

DMS: differentially methylated CpG-sites
CNS: central nervous system
EDSS: expanded disability status scale
HLA: human leukocyte antigen; MS, multiple sclerosis
PBMC: peripheral blood mononuclear cells
PPMS: primary-progressive multiple sclerosis
RRMS: relapse-remitting multiple sclerosis
